# Immediate and delayed hypersensitivity to biologicals in children—A practical approach: An EAACI task force report

**DOI:** 10.1111/pai.70277

**Published:** 2026-04-05

**Authors:** Francesca Mori, Marina Atanaskovic‐Markovic, Annick Barbaud, Sevim Bavbek, Jean‐Christoph Caubet, Leticia de las Vecillas, Mattia Giovannini, Ekaterina Khaleva, Marina Labella, Luis Moral, Carmen Riggioni, Vito Sabato, Sophia Tsabouri, Stefania Arasi

**Affiliations:** ^1^ Allergy Unit Meyer Children's Hospital IRCCS Florence Italy; ^2^ University Children's Hospital of Belgrade Belgrade Serbia; ^3^ Faculty of Medicine University of Belgrade Belgrade Serbia; ^4^ Sante Sorbonne Université, INSERM, Institut Pierre Louis d'Epidémiologie et de Santé Publique, AP‐HP. Sorbonne Université Paris France; ^5^ Département de Dermatologie et Allergologie Hôpital Tenon Paris France; ^6^ Department of Chest Disease, Division of Immunology and Allergy Ankara University School of Medicine Ankara Türkiye; ^7^ Pediatric Allergy Unit, Department of Pediatrics, Gynecology and Obstetrics University Hospitals of Geneva Geneva Switzerland; ^8^ Department of Allergy La Paz University Hospital Madrid Spain; ^9^ La Paz University Hospital Research Institute (IdiPAZ) Madrid Spain; ^10^ Department of Health Sciences University of Florence Florence Italy; ^11^ Human Development and Health, Faculty of Medicine University of Southampton Southampton UK; ^12^ Royal Hampshire County Hospital Winchester UK; ^13^ Allergy Research Group Instituto de Investigación Biomédica de Málaga y Plataforma en Nanomedicina‐IBIMA Plataforma BIONAND Málaga Spain; ^14^ Allergy Unit Hospital Regional Universitario de Málaga Málaga Spain; ^15^ Pediatric Allergy and Respiratory Unit Dr. Balmis General University Hospital Alicante Spain; ^16^ Alicante Institute for Health and Biomedical Research (ISABIAL) Alicante Spain; ^17^ Division of Immunology and Allergy The Hospital for Sick Children and the SickKids Food Allergy and Anaphylaxis Program Toronto Ontario Canada; ^18^ Department of Paediatrics Temerty Faculty of Medicine, University of Toronto Toronto Ontario Canada; ^19^ Department of Immunology, Allergology and Rheumatology University of Antwerp Antwerpen Belgium; ^20^ Department of Immunology, Allergology and Rheumatology University Hospital Antwerp Edegem, Antwerp Belgium; ^21^ Faculty of Medicine, School of Health Sciences University of Ioannina Ioannina Greece; ^22^ Translational Research in Pediatric Specialties Area, Division of Allergy Bambino Gesù Children's Hospital, IRCCS Rome Italy

**Keywords:** biologicals, children, delayed, hypersensitivity, immediate

## Abstract

In the era of precision medicine, biologicals demonstrate how therapies can be personalized and directed against new targets. This type of therapy includes different molecules such as growth factors, immune modulators, vaccines, and monoclonal antibodies (mAbs). In recent years, biologicals have been increasingly developed and authorized, although their use in children is limited compared to that in adults, due to the complexity of the pharmacokinetics and pharmacodynamics of the involved proteins, as well as other factors, such as regulations governing clinical trials. Regardless, biologicals are used with efficacy in children to treat various diseases, including oncological, hematological, atopic, and rheumatological diseases. In parallel with the increased use of biologicals, there has been an increase in the unwanted effects of these agents. This paper aims to provide physicians with a practical approach to differentiate between the types of reactions to biologicals in children, especially mAbs, based on the frequency of use, for a comprehensive allergy workup. Starting from a clinical case (i.e., phenotype), specific biomarkers of the involved molecular mechanism (i.e., endotype) are described, providing the reader with currently known instruments to guide the diagnosis. Finally, practical limitations, preventive measures, and unmet needs were discussed by a panel of experts.

**Key message**

Hypersensitivity reactions to biologicals are increasingly reported; however, pediatric data on their immunopathogenesis, diagnosis, and management remain limited. This review summarizes the current evidence on clinical phenotypes, underlying mechanisms, and practical diagnostic and therapeutic strategies in children, highlighting the central role of allergy workup and tailored approaches such as premedication, drug provocation testing, and desensitization.

## 1 INTRODUCTION

In the era of precision medicine, biologicals demonstrate how therapies can be personalized and directed against new targets. This type of therapy includes different molecules such as growth factors, immune modulators, vaccines, and monoclonal antibodies (mAbs).^1^, ^2^

In recent years, biologicals have been increasingly developed and authorized, although their use in children is limited compared to that in adults, due to the complexity of the pharmacokinetics and pharmacodynamics of the involved proteins, as well as other factors, such as regulations governing clinical trials. Regardless, biologicals are used with efficacy in children to treat various diseases, including oncological, hematological, atopic, and rheumatological diseases. In parallel with the increased use of biologicals, there has been an increase in the unwanted effects of these agents.

Biologicals have the potential to elicit immune responses, resulting in the production of anti‐drug antibodies (ADA).^3^ Consequently, loss of response to the biological agents and/or infusion reactions may occur, limiting treatment efficacy.

Humoral responses can be mediated by IgE or non‐IgE ADA isotypes and are partially due to the residual immunogenicity of biologicals, which remains despite a progressive increase in human sequences in their structure. Immediate reactions may also be due to the release of cytokines, such as infusion‐related reactions (IRRs) and cytokine release reactions (CRRs).^4^ The group of immediate reactions also includes the mixed reactions in which both humoral and cytokine‐driven mechanisms are involved. Cell‐driven reactions are delayed type IV reactions, which include maculopapular exanthemas (MPEs) and, less frequently, severe cutaneous adverse reactions (SCARs). Type III reactions to biologicals are primarily characterized by serum sickness (SS) or serum sickness‐like reactions (SSLRs), which are also observed in children. This model, which is based on the chronology and effector cells involved, has been updated to include endotypes, phenotypes, and biomarkers in the more recent EAACI classification.^5^

Owing to the limited data available, it is difficult to compare the rate of hypersensitivity reactions (HSRs) between adults and children. However, the occurrence appears to be similar, ranging from approximately 4% to 20% depending on the specific drug.^6^ Local reactions to anti‐interleukin (IL)1 therapy and anaphylaxis to rituximab and tocilizumab are among the most frequently reported reactions.^7^ In addition, delayed severe reactions induced by biologicals in children have also been reported.^8^ As in adults, HSRs to biologicals may negatively affect disease progression and patients' quality of life.

**FIGURE 1:**
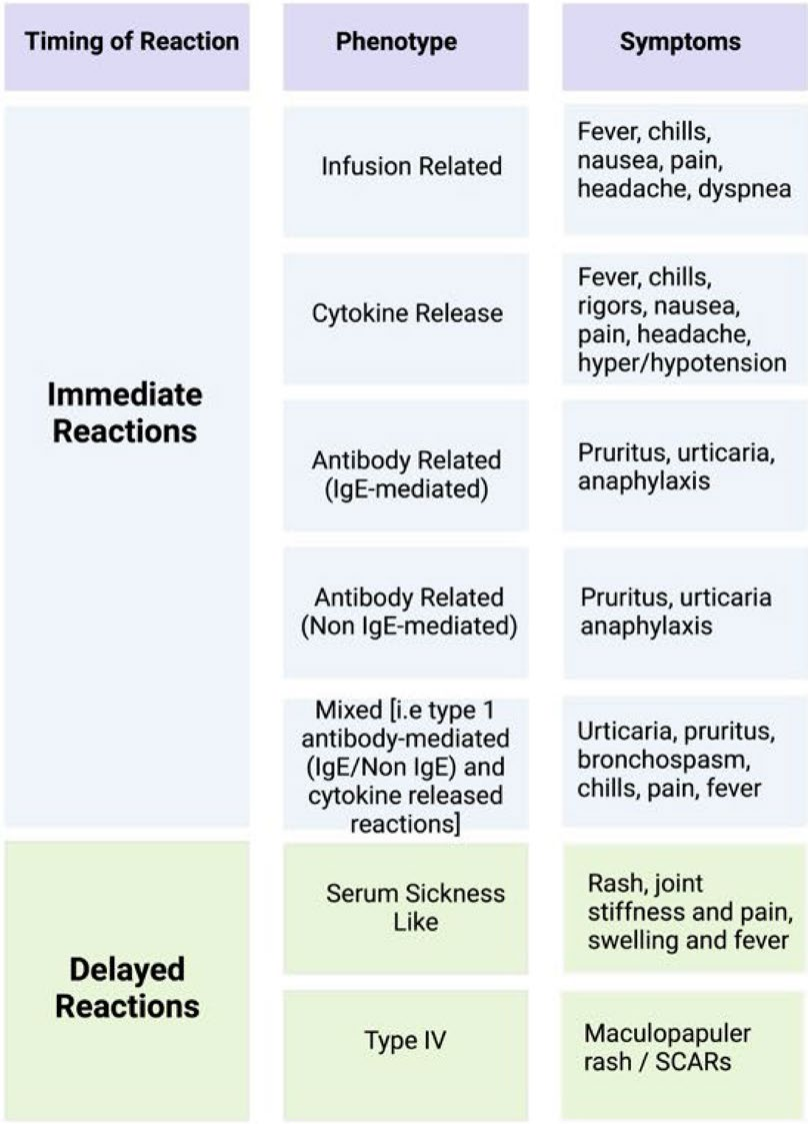
Types of reactions to biologicals in children.

This paper aims to provide physicians with a practical approach to differentiate between the types of reactions to biologicals in children, especially mAbs, based on the frequency of use, for a comprehensive allergy workup (Figure 1). Starting from a clinical case (i.e., phenotype), specific biomarkers of the involved molecular mechanism (i.e., endotype) are described, providing the reader with currently known instruments to guide the diagnosis. Finally, practical limitations, preventive measures, and unmet needs were discussed by a panel of experts.

## 2 IMMEDIATE REACTIONS

### 2.1 Infusion‐related reactions (IRRs)

Box 1.

BOX 1 **Clinical Case**

A 15‐year‐old female was brought to the emergency department complaining of abdominal pain, diarrhea, and bloody stools. In the past month, she had 4 kg weight loss and fatigue. Upon physical examination, her abdomen was distended and tender in the lower left quadrant. Blood tests reveal anemia, elevated C‐reactive protein and erythrocyte sedimentation rate. A colonoscopy showed mucosal ulcers in a continuous pattern starting from the rectum. A biopsy confirmed chronic inflammation of the colon lining. She was diagnosed with moderate to severe ulcerative colitis and started treatment with infliximab. Fifteen minutes after starting the first infliximab infusion, she presented flushing, fever, chills and body pain. The infusion rate was reduced, and paracetamol was administered, which alleviated the symptoms and allowed for the administration of the drug. Subsequent infusions were tolerated at a lower infusion rate and with premedication consisting of paracetamol. The final diagnosis was IRR to infliximab.

#### 2.1.1 Clinical presentation

IRR is defined as an adverse reaction to the infusion of pharmacological or biological substances that potentially occurs on the first day of drug administration.^9^ The World Health Organization (WHO) nomenclature classifies IRRs into major subtypes according to the time interval between the infusion and the onset of symptoms. Reactions that develop during infusion or within 1–2 h of completion are labeled as immediate‐type reactions.^10^ Other organizations extended this period up to six hours.^11^, ^12^ They primarily manifest during the initial infusion(s) and vary widely from mild to life‐threatening adverse events. The most common IRRs are mild to moderate and include flushing, fever, chills, rigors, nausea, pain, and headache. Generally, IRRs are less severe than a cytokine storm, such as in the CRRs. Moreover, the difference between IRRs and CRRs lies in the self‐limiting nature of IRRs, which occurs after repeated exposure following a reduction in the infusion rate and/or the inclusion of specific premedication. More severe reactions with systemic symptoms were less frequently observed.^13^, ^14^, ^15^

#### 2.1.2 Immunopathogenesis

Both immediate immune and non‐immune‐mediated reactions to biologicals share similar clinical manifestations and mechanisms, making it challenging to pinpoint the precise nature of the immune response.^12^, ^16^ In some of these immediate reactions, an IgE‐mediated mechanism could be involved in the release of tryptase as a biomarker for differentiating Type I reactions.^1^ For non‐IgE immediate reactions, for example, the lymphocytes release tumor necrosis factor alpha (TNF‐α), IL‐6, and IL‐1β, which could be detected, differentiating CRRs.^17^

#### 2.1.3 Diagnosis

Diagnosis is based on the clinical history and evolution after specific preventive measures as lowering the infusion rate and including premedication. Skin tests are not useful.^14^

#### 2.1.4 Management and treatment

IRRs are self‐limited can become less severe on repeated exposures^18^ and are typically managed by anti‐inflammatory/anti‐allergic premedication and by decreasing the infusion rate.^17^ In milder cases, the infusion rate should be slowered until the patient is asymptomatic. Once stable, it can be progressively increased up to the maximum tolerated infusion speed. In severe cases, infusion should be stopped, and the symptoms are treated accordingly. Therapeutic recommendations are mainly based on case reports and expert opinions.^12^ For subsequent infusions, a slower infusion rate may prevent relapse associated with premedication, which could include paracetamol, Non‐Steroidal Anti‐Inflammatory Drugs (NSAIDs), antihistamines, and/or steroids.^1^

### 2.2 1‐ Cytokine Release Reactions (CRRs)

Box 2.

BOX 2 **Clinical Case**

A 6‐year‐old girl was diagnosed with Granulomatosis with Polyangiitis and started induction treatment on rituximab weekly. Twenty minutes after the first infusion was completed, the patient presented with chills, back pain, cold, and rigors. The clinical examination was otherwise normal, and vital signs revealed fever (38°C). Intravenous (IV) fluids and paracetamol were administered, and symptoms resolved in half an hour.

IL‐6 and tryptase blood levels were withdrawn 30 minutes after the reaction, showing values of 1.030 pg/mL for IL‐6 and 1.4 ng/mL for tryptase. Baseline levels were 3 pg/mL and 1.2 ng/mL, respectively. Skin tests with rituximab were negative. Based on clinical features and IL‐6 mediator's level, the diagnosis of CRR‐phenotype induced by rituximab was confirmed. The patient is currently receiving rituximab with desensitization (3 dilutions and 12‐step protocol) with good tolerance.

#### 2.2.1 Clinical presentation

CRRs can occur during first‐lifetime exposure or after repeated exposures. The main clinical manifestations include musculoskeletal, cardiovascular, and constitutional symptoms (i.e., generalized illness not representative of any specific organ system or syndrome such as weight loss, fever, and fatigue).^18^ They can range from mild to moderate symptoms (e.g., chills, rigors, flushing, fever, nausea, vomiting, rash, back pain, hypertension, and headache) to severe life‐threatening symptoms (e.g., hypoxia, hypotension, heart, renal or liver failure, disseminated intravascular coagulopathy, and neurological dysfunction).^6^, ^19^

Regarding time onset, although CRRs have been classified as immediate using this practical approach, it is important to mention that it can also appear more than 6 hours or even days after the infusion, posing a real challenge for the differential diagnosis of sepsis when the patient presents to the emergency room in the acute phase.^20^

#### 2.2.2 Immunopathogenesis

The pathophysiology is not completely understood, but the hypothesized mechanism is that the release of high levels of proinflammatory cytokines may originate from multiple cellular sources, including monocytes, macrophages, and T lymphocytes.^21^ This activates the inflammatory cascade and causes a systemic inflammatory response that can induce multiple organ failure.^22^

#### 2.2.3 Diagnosis

The suspicion of this phenotype is based on the clinical characteristics and onset of the reaction, which differs from those of other phenotypes. Although distinguishing CRRs from IRRs can be challenging, the latter are self‐limited after several exposures and respond to premedication and low infusion rates.^6^

Skin testing is not useful to confirm a CRR phenotype; however, in vitro tests can show an increase in TNF‐α and IL‐6 blood levels during the reaction compared to baseline values. IL‐6 has been identified as an important biomarker for the verification of this phenotype‐endotype.^18^, ^23^, ^24^ Although no specific cut‐off value has been standardized, an elevation of 40 times compared to the baseline has been proposed to identify a massive release of cytokines.^24^, ^25^ Moreover, IL‐6 is associated with a clinical picture that includes thermal and hemodynamic changes accompanied by constitutional, cardiovascular, and neuromuscular symptoms.^24^ There is no increase in tryptase levels.

#### 2.2.4 Management and treatment

The management of CRRs includes both short‐ and long‐term treatments. In the acute phase, symptoms should be treated according to the last EAACI position paper on the management of drug‐induced anaphylaxis in children.^26^ However, there are some specific aspects of the CRR‐phenotype. Fluids may be beneficial by decreasing the concentration of antigens and mediators, thereby facilitating the rapid clearance of immunogenic substances.^19^, ^27^ Aspirin can be considered for children when flushing, and paracetamol can be used to treat fever, chills, and pain.^19^

Long‐term treatment focuses on preventing reactions by safely administering the culprit drug when it is the first choice for the patient.^26^, ^28^, ^29^ Most published case series that included CRR patients were adults; however, some recent articles have described CRRs induced by rituximab and infliximab in children.^30^ In general, the indications for desensitization are the same in adults and children; however, young children may not be able to report the initial mild and subjective symptoms of a reaction, which therefore require special attention in this population during the procedure.^31^, ^32^, ^33^, ^34^ Although desensitization is best demonstrated for IgE‐mediated reactions mechanistically, there is a place for its use in patients who experience severe CRRs.^19^, ^35^

### 2.3 2‐Type I Antibody‐Mediated Reactions (IgE/non‐IgE)

Box 3.

BOX 3 **Clinical Case**

A child diagnosed with Crohn's disease, aged 5 years and 11 months, came to the allergy unit for examination of a suspected HSR to infliximab. About 20 min after receiving the 13th dose of infliximab, the child developed difficulty breathing, hypersalivation, facial swelling, vomiting, generalized urticaria, and then hypotension (blood pressure <60 mmHg). After the administration of adrenaline, antihistamine, and steroid, the symptoms disappeared. Increased levels of serum tryptase confirmed that the child had anaphylaxis. Four weeks after the reaction, an allergy workup for infliximab was performed. The skin prick test with infliximab was 3 mm in diameter, as the positive control (histamine). The negative control (0.9% saline solution) was negative. An intradermal test was performed, and the immediate reading at 20 min was considered positive. Based on the clinical presentation, a Type I IgE‐mediated anaphylaxis is a consistent diagnosis. Since infliximab is the drug of choice for the treatment of Crohn's disease and there is currently no substitute, infliximab desensitization was performed with 3 dilutions and 12 steps protocol. Desensitization was completed successfully without adverse reactions; no premedication (steroids and antihistamines) was needed. Desensitization with infliximab was performed every 4 weeks and it was well tolerated during subsequent cycles.

#### 2.3.1 Clinical presentation

Type I antibody‐dependent anaphylaxis usually occurs after at least one exposure to the culprit drug. Urticaria is the most frequent skin manifestation but respiratory and gastrointesional symptoms can appear. The severity can vary from mild reactions to severe anaphylaxis including heamodinamic involvement.

#### 2.3.2 Immunopathogenesis

Data from the Food and Drug Administration (FDA) Adverse Event Reporting System (FAERS) showed that biologicals, especially mAbs, appeared to be one of the most common drugs associated with anaphylaxis.^36^ All biologicals have inherent immunogenicity and potential to induce ADAs.^37^ Type I antibody‐dependent anaphylaxis involves IgE‐ and IgG‐mediated reactions.^38^ IgE development is the result of a Th2‐skewed cellular immune response against biologicals, as demonstrated by sIgE to rituximab and infliximab.^2^, ^39^ Typically, IgE‐mediated reactions require several exposures before occurrence, although there are exceptions such as cetuximab, which can cause IgE‐mediated reactions on the first exposure due to preformed anti‐alpha Gal IgE antibodies.^2^, ^39^, ^40^, ^41^

All biologicals may potentially induce the development of ADAs; however, several factors can influence their immunogenicity. These include the underlying disease to be treated, the patient's immune status, other concomitant medications, and drug‐related factors, such as the degree of humanization, glycosylation pattern, type of sourced cells, dosing interval, and excipients with allergenic potential, as well as the route of administration.^2^, ^39^, ^42^ The culprit drug infliximab, a chimeric mAb, is one of the most frequently reported biologicals for anaphylactic reactions.^9^

#### 2.3.3 Diagnosis

Both in vivo and in vitro tests are used to diagnose anaphylaxis. Positive skin testing, essentially an immediate reading intradermal test (IDT), have been reported in patients with a history of anaphylaxis to several biologicals, including rituximab, anti‐TNF agents, brentuximab, and trastuzumab.^10^, ^11^, ^12^ Positive results from skin testing may differentiate between IgE‐ and non‐IgE‐mediated reactions, but the lack of standardization of skin tests with biologicals, as well as the possibility of false positives and false negatives, should be kept in mind.^2^

Basophil activation test (BAT) is a complementary method used to diagnose immediate IgE‐mediated reactions. It is based on the evaluation of CD63 and CD203 as surface markers of basophil activation. Furthermore, additional studies with larger patient and child populations are needed to establish the routine use of BAT in diagnosing immediate HSRs to biological agents.^43^, ^44^ The drug provocation test (DPT) is the gold standard for diagnosing of HSRs to biological agents.^45^ The detection of ADA, either IgE or IgG, is currently the most feasible approach for assessing immunogenicity.^16^ Human data on IgG‐mediated anaphylaxis are limited to neuromuscular blocking agents and, in most cases, are indirect and lag significantly behind animal model findings.^46^ Detectable levels of anti‐infliximab IgM antibodies were detected in three adults with severe infliximab anaphylactic reactions.^39^ There are commercially available tests for the assay of non‐isotype‐specific ADA, but the lack of commercially available tests for IgE ADA detection represents a crucial unmet need in the diagnostic workup of immediate HSRs to biologicals.^2^, ^37^, ^42^

#### 2.3.4 Management and treatment

As an allergist, initial reactions are rare, but should be promptly treated. In the absence of an effective and safe alternative to the culprit drug, rapid drug desensitization is performed, which is an effective management strategy for safely administering a medication that causes anaphylaxis by providing temporary immune tolerance to drugs to which patients present with anaphylaxis.^2^, ^41^, ^42^, ^47^, ^48^, ^49^, ^50^, ^51^ Owing to the loss of tolerance after the administration, desensitization may be repeated in each treatment cycle.

### 2.4 3‐Mixed Reactions

Box 4.

BOX 4 **Clinical Case**

A 13‐year‐old male was admitted to the pediatric hematology clinic for a low‐grade B‐cell (follicular) non‐Hodgkin lymphoma. It was decided to treat him with 6 cycles of R‐CHOP (rituximab, cyclophosphamide, adriamycin, vincristine and prednisone protocol). Since the first infusion, the patient developed flushing and pruritus within 5 minutes of administration. The infusion was interrupted, and IV pheniramine was administered. The infusion was slowly restarted, but after 15 min, he reacted with fever, generalized urticaria, dyspnea, nausea and severe back pain. His physical examination revealed 39.2°C body temperature, arterial blood pressure of 110/60 mmHg (within normal range for the patient), arterial O_2_ saturation: 92%, 120 beats/min tachycardia, and bilateral rhonchi with no stridor or pharyngeal oedema. The infusion was stopped, and the reaction was treated accordingly. The tryptase level was 20.40 𝜇g/L (𝑛: <11 𝜇g/L) at 1.5 h, and the IL‐6 level was 203 pg/mL after the HSR. Skin tests, such as prick and IDT to rituximab, were positive 4 weeks after the index reaction.^21^A mixed reaction to rituximab was diagnosed.

#### 2.4.1 Clinical presentation

There is increasing recognition that some biologicals may produce mixed reactions.^18^ They occur during the administration of the biological agent or immediately after administration or infusion.^6^

#### 2.4.2 Immunopathogenesis

Immunopathogenesis combines the immunological routes implicated in Type I (IgE or non‐IgE) and CRR. Mast cells, basophils, neutrophils, macrophages, and T‐cells are implicated in the underlying mechanisms of this type of reaction.^6^

In the adult population, they are estimated to account for up to 20% of the reactions to biologicals.^18^ In children, mixed reactions have been reported with rituximab, infliximab, and adalimumab.^30^

#### 2.4.3 Diagnosis

Accurate diagnosis of mixed reactions requires a combination of clinical manifestations and biomarkers, including those implicated in Type I reactions and CRRs. Therefore, a positive skin test and/or a specific IgE to the implicated biological drug (directly detected or through a positive BAT) can be found. Regarding serum biomarkers, the patient can present with acute elevation of tryptase but also increased levels of several cytokines such as IL‐6, IL‐1, and TNF‐α.^2^, ^4^, ^6^

#### 2.4.4 Management and treatment

The acute management of mixed reactions includes symptomatic treatment. Based on the clinical presentation and biomarker results, the reaction evaluation will determine drug reintroduction following the same pathways as in Type I reactions and CRRs.^2^

Considering the grade of the initial reaction, specific symptoms, and biomarker results, a challenge or desensitization will be proposed to maintain treatment in case no similar alternatives exist for the patient.^6^ Desensitization has been proven effective in preventing this type of reaction to biologicals.^18^, ^30^

## 3 DELAYED HYPERSENSITIVITY REACTIONS TO BIOLOGICALS

### 3.1 1‐Serum Sickness (SS)/Serum Sickness‐Like Reactions (SSLRs)

Box 5.

BOX 5 **Clinical Case**

A case of rituximab‐induced SS was reported in a 6‐year‐old boy diagnosed with nephrotic syndrome. He was premedicated with IV chlorphenoxamine and IV paracetamol before its administration, and after the first dose of the treatment, no immediate infusion reactions occurred. However, 8 days after the first infusion, the patient suffered from myalgia and arthralgia, which spontaneously improved within 24 h. Two months later, a second rituximab dose was administered, and he suffered from arthralgia and a diffuse rash the following week, which disappeared without any intervention within 2 days. Three weeks later, a third rituximab dose was administered, and the patient suffered from mild fever, generalized erythematous rash, choking sensation, and swelling of lips and periorbital regions during the infusion of the drug, which was discontinued. Subsequently, the patient was treated with methylprednisolone and adrenaline with quick recovery. The first and second reactions were diagnosed as SS, and the third one as anaphylaxis to rituximab, which was then discontinued.^52^

#### 3.1.1 Clinical presentation

SS involves immune activation against agents that can cause specific clinical manifestations after 7–21 days. The symptoms include fever, rash, and polyarthralgia or arthritis, myalgia, malaise, fatigue, conjunctival hyperemia, and purpura.^53^ SS is most frequently observed in adults. SSLRs are sometimes confused with SS because they occur within a similar time course and share some common manifestations, even if they are not characterized by the same laboratory findings.

Both children and adults can be affected by these clinical entities, although SSLR has been more commonly observed in association with beta‐lactam antibiotics in children.^54^, ^55^

A case of omalizumab‐induced SSLR was reported in a 12‐year‐old girl diagnosed with angioedema and chronic urticaria. One week after the second administration, she experienced headache, joint pain, malaise, and a morbilliform rash without reactions at the site of administration. Despite this, the patient continued the treatment for 6 months, during which she suffered from joint pain, significant axillary and cervical lymphadenopathy, worsening malaise, and mild thrombocytopenia after each administration. Subsequently, she was diagnosed with SSLR, omalizumab was discontinued, and the clinical manifestations resolved.^56^ Furthermore, SSLRs to omalizumab has been reported in adult patients.^57^, ^58^

Finally, several other biologicals, including rituximab,^53^, ^59^, ^60^, ^61^, ^62^, ^63^ dupilumab,^64^, ^65^ infliximab,^66^ ixekizumab,^67^ and obinutuzumab have been associated with SS in adults.^68^

#### 3.1.2 Immunopathogenesis

SS represents an immune response against a culprit agent mediated by complement‐fixing IgM and IgG antibodies directed toward an immunogenic portion of a trigger (type III HSR) with neutrophil recruitment.^53^ Several culprit biologicals and routes of administration are associated with SS in children and adults.

The pathophysiology of SSLR is unknown; however, it is not associated with circulating immune complexes, nephrotoxicity, vasculitis, or depletion of complement as in SS.^69^

### 3.2 Diagnosis

Clinical manifestations and timing are the cornerstones of the diagnosis of SS. Organ impairment has been reported in more severe cases.^53^ There is no evidences on the utility of skin biopsies in case of SS. Regarding SSLR, a quick recovery after drug discontinuation or after steroid treatment, has been reported.^53^

#### 3.2.1 Management and treatment

Once the reaction is identified, the interruption of the culprit agent is crucial for symptoms resolution. Systemic steroids are the mainstay of SS treatment together with drugs targeting clinical manifestations, such as paracetamol and antihistamines. Even if the reactions can resolve after drug discontinuation, a case of SS associated with anaphylaxis after re‐exposure to rituximab has been reported, with the need for adrenaline due to hemodynamic repercussion.^53^ Re‐administration of the culprit biological after an SS reaction is not indicated.^2^

### 3.3 2‐Type IV Reactions

Box 6.

BOX 6 **Clinical Case**

A 17‐year‐old Chinese female suffering from severe atopic dermatitis, the second day after the first injection of dupilumab (600 mg), developed a diffuse cutaneous rash, rapidly progressing from the face to her neck, flexure joints of the upper limbs, and dorsum of the hands.

The rash was characterized by numerous non‐follicular, pinhead‐sized pustules on diffuse edematous and erythematous plaques. She had fever (38.5°C) without any involvement of organs or mucous membranes. Laboratory investigation revealed elevated neutrophils (9100 cells/mm^3^), eosinophils (9.5%, normal range 0.4%–8.0%), and C‐reactive protein (13.4 mg/L). Liver and kidney function tests were normal. Concomitant infectious diseases were excluded. The results of laboratory exams and clinical history were consistent with the diagnosis of acute generalized exanthematous pustulosis (AGEP).^70^ According to the European Study of Severe Cutaneous Adverse Reactions (EuroSCAR) scoring system, the final score was 9, and the diagnosis of AGEP was definite. Dupilumab was discontinued and the patient was treated with systemic drugs (azithromycin, compound glycyrrhizin potassium chloride and antihistamines) and topical tacrolimus 0.1% ointment. Rash progression promptly stopped, and skin lesions improved after 2 weeks with complete resolution 1 month later.^71^

#### 3.3.1 Clinical presentation

Data on type IV hypersensitivity reactions in children are scarce. Therefore, most data are from adult studies.

Clinical presentations include injection site reactions (ISR), MPE, eczematous exanthema, delayed urticaria and, rarely, SCAR such as AGEP, Drug Rash with Eosinophilia and Systemic Symptoms (DRESS), Stevens–Johnson syndrome (SJS), and toxic epidermal necrolysis (TEN).

ISR, appearing in 24–48 h and lasting for several days, is characterized by an infiltrated erythema associated with an itch or burning sensation. ISRs are common complications of subcutaneous biologicals and do not always display the features of delayed HSRs. Most ISRs appear within the first weeks of treatment and tend to disappear over time.

#### 3.3.2 Pathogenesis

The pathogenesis of ISRs is not fully understood. Some could be due to irritation, whereas others, with a recall phenomenon and delayed positive reactions to intradermal tests, could be due to specific T cell mediated reactions.

Systemic reactions, from MPEs to SCARs, are classified as by a type IV HSRs. This model, which is based on the chronology and effector cells involved, has been updated to include endotypes, phenotypes, and biomarkers in the more recent EAACI classification.^5^ DRESS to anti IL‐1 or anti IL‐6 seems to be strongly associated with HLA‐DRB1*15 haplotypes.

It is essential to note that some generalized skin reactions can result from the pharmacological effects of biologicals.

#### 3.3.3 Diagnosis

Most SCARS are diagnosed using different scoring systems (RegiSCAR for DRESS; ALDEN Scale for drug causality assessment in SJS/TEN, and EuroSCAR for AGEP). However, these scoring systems are used in adults and have not been validated in children.

Biopsies are frequently not useful as diagnostic tools, even though they are often performed in adults with SCARs, particularly for the diagnosis of AGEP. Euroscar AGEP includes histopathological criteria, and biopsies are specific to subcorneal pustules.

For delayed HSRs, the values of in vivo skin tests with delayed readings and in vitro tests are unknown.

#### 3.3.4 Management and treatment

Acute management includes discontinuation of the drug and, depending on the severity of the clinical manifestations, topical, or systemic steroids. In the case of SCAR, the use of other immunomodulators such as cyclosporine and supportive therapies can be needed. In cases of non‐immediate systemic reactions, other factors such as infections should always be considered in the differential diagnosis. Immune deviation can also be involved. In the case of ISRs, especially without the recall phenomenon, the treatment can be continued. For mild/moderate MPEs, drug reintroduction (DPT, desensitization) may be considered based on the necessity of treatment. However, there is a lack of standardized protocols, especially for children. In case of SCARs, drug reintroduction is contraindicated.^2^, ^6^, ^18^, ^72^, ^73^, ^74^

## 4 PRACTICAL LIMITATIONS

Despite these limitations, tryptase serum levels are the most studied biomarker to identify mast cell activation‐driven reactions, such as ADA‐mediated and mixed reactions. It is especially useful to measure it during severe reactions when its elevation is more striking, with a high positive predictive value, but a low negative predictive value.^75^, ^76^, ^77^ To detect tryptase elevation, it is crucial to compare the reaction value with the patient's baseline level.

In the case of IL‐6, a large elevation has been detected during CRRs.^22^, ^23^ For a better understanding of the underlying immune mechanism of the reaction to establish a more tailored management, we recommend measuring both tryptase and IL‐6 levels.

ADA detection and cytokine analysis have not been widely performed, and not commercially available. BAT is not always available, either. The price of these drugs also limits their use for in vivo and in vitro allergy testing resulting in expensive diagnostic workup. Companies could contribute avoiding this last aspect by commercializing small aliquots for allergy diagnostic tests use.

## 5 PREVENTING MEASURES

In case of IR, primary prevention may be based on lowering the first infusion rate and/or by using premedication. Secondary prevention may require desensitization. To mitigate ADA‐mediated reactions, risk factors should be identified. ADA testing can be performed to identify ADA‐induced immunogenicity.

## 6 UNMET NEEDS

The occurrence of HSRs to biologicals has been increasing in recent years, especially for mAbs, based on their frequency of use. However, limited data are available on pediatric age for both diagnosis and treatment.^7^, ^8^, ^34^, ^78^ The development of unwanted effects represents a relevant clinical concern and challenge for the use of biologicals in daily practice. This paper provides up‐to‐date, practical information on the pediatric clinical manifestations, pathogenesis, diagnosis, and management of children with immediate and delayed hypersensitivity to biologicals.

The underlying immunological mechanisms vary, and physicians should be aware of their occurrence and able to recognize them through their clinical presentation. Currently, there is still limited knowledge of the endotypes and specific biomarkers; however, allergy workup, including skin testing and the measurement of some biomarkers such as tryptase and IL‐6, constitutes the main tool to establish appropriate management and plan the drug reintroduction.

According to the classification, there are gray zones; for example, it is unclear whether the CRRs and IRRs are part of the same spectrum of reactions. No systematic studies have compared the use of premedication in the management of these reactions.

Generally, IRRs benefit from lowering the infusion rate and premedication when are mild in severity. However, to prevent more severe reactions such as CRRs, a desensitization protocol may be applied when reintroducing the drug. In ADA‐mediated desensitization, it is mandatory to keep the treatment in a safe and effective manner. In contrast, the role of premedication in delayed reactions remains debatable.

Regarding diagnosis, in vitro testings needs validation in children, and there is no recommendation about the safe choice of non‐cross‐reactive biologicals.

DPT is the gold standard for diagnosing drug hypersensitivity. However, only expert drug allergy centers can perform it when no other diagnostic tools have provided conclusive results and, for example, the clinical history is not clear.^45^

## 7 CONCLUSION

**FIGURE 2:**
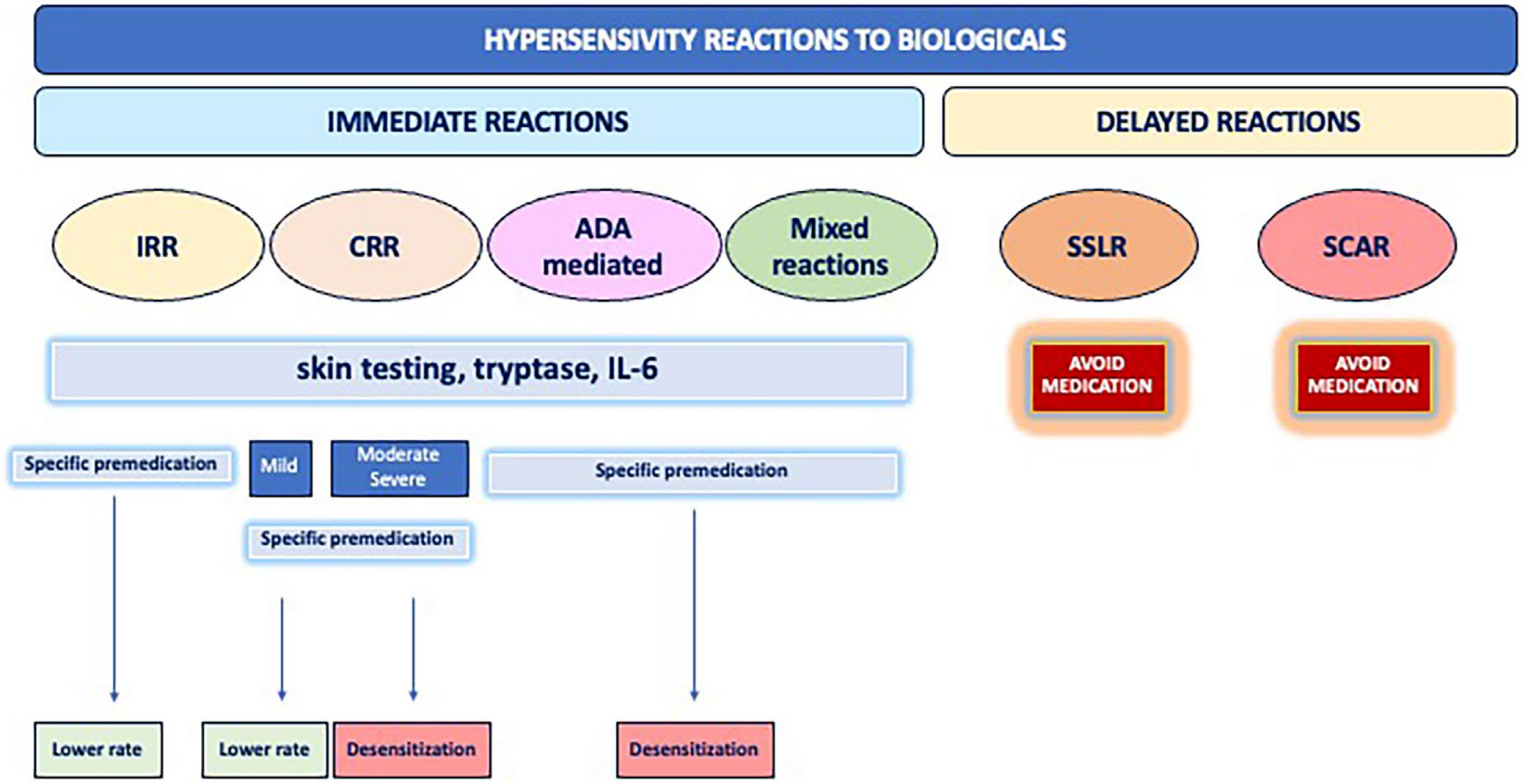
Management of reactions to biologicals in children.

For biologicals, the panel of experts suggests the following (Figure 2): DPT if the initial reaction is immediate, mild with negative tests; desensitization in the case of moderate to severe, immediate reactions and in the absence of an equally effective biological agent; non‐reintroducing the culprit biological agent in the case of SCARs or SS; tailored management can be carried out patient‐by‐patient to deepen the molecular mechanism underlying HSRs's clinical presentation.

Both DPT and desensitization should be performed in safely by trained personnel in experienced drug HSRs's centers. For all the above‐mentioned reasons, reactions to biologicals require a multidisciplinary management, involving referral specialists (i.e., oncologists, hematologists, rheumatologists, immunologists, gastroenterologists, pediatricians, dermatologists), the allergists and the pharmacists.

## AUTHOR CONTRIBUTIONS

**Francesca Mori:** Conceptualization; methodology; writing – original draft; writing – review and editing; data curation; formal analysis. **Marina Atanaskovic‐Markovic:** Writing – original draft; writing – review and editing. **Annick Barbaud:** Writing – original draft; writing – review and editing. **Sevim Bavbek:** Writing – original draft; writing – review and editing. **Jean Cristoph Caubet:** Writing – original draft; writing – review and editing. **Leticia de las Vecillas:** Writing – original draft; writing – review and editing. **Mattia Giovannini:** Writing – original draft; writing – review and editing. **Ekaterina Khaleva:** Writing – original draft; writing – review and editing. **Marina Labella:** Writing – original draft; writing – review and editing. **Luis Moral:** Writing – original draft; writing – review and editing. **Carmen Riggioni:** Writing – original draft; writing – review and editing. **Vito Sabato:** Writing – original draft; writing – review and editing. **Sophia Tsabouri:** Writing – original draft; writing – review and editing. **Stefania Arasi:** Writing – original draft; writing – review and editing; supervision.

## FUNDING INFORMATION

This Task force report was supported by the European Academy of Allergy and Clinical Immunology (EAACI) under the EAACI Task Force Immediate and delayed hypersensitivity to biologicals in children – a practical approach DAIG & Pediatric Section, 40708, 2024.

## CONFLICT OF INTEREST STATEMENT

SA declares to have received consulting fees as advisory board member for Aimmune, Allergy Therapeutics and Novartis, as speaker/discussant for Thermo Fischer, Ulrich, DBV, and Stallergenes Greer outside the submitted work. MG reports personal fees from Sanofi, Thermo Fisher Scientific. CR is an associate editor for PAI journal and has received ad board fees for ALK and speaker fees from Thermo Fisher.
